# *miRsig*: a consensus-based network inference methodology to identify pan-cancer miRNA-miRNA interaction signatures

**DOI:** 10.1038/srep39684

**Published:** 2017-01-03

**Authors:** Joseph J. Nalluri, Debmalya Barh, Vasco Azevedo, Preetam Ghosh

**Affiliations:** 1 Department of Computer Science, School of Engineering, Virginia Commonwealth University, Richmond, Virginia,USA; 2 Center for Genomics and Applied Gene Technology, Institute of Integrative Omics and Applied Biotechnology, Purba Medinipur, West Bengal, India; 3 Laboratório de Genética Celular e Molecular, Departamento de Biologia Geral, Instituto de Ciências Biológicas (ICB), Universidade Federal de Minas Gerais, Pampulha, Belo Horizonte, Minas Gerais, Brazil; 4 Xcode Life Sciences, 3D Eldorado, 112 Nungambakkam High Road, Nungambakkam, Chennai, Tamil Nadu-600034, India

## Abstract

Decoding the patterns of miRNA regulation in diseases are important to properly realize its potential in diagnostic, prog- nostic, and therapeutic applications. Only a handful of studies computationally predict possible miRNA-miRNA interactions; hence, such interactions require a thorough investigation to understand their role in disease progression. In this paper, we design a novel computational pipeline to predict the common signature/core sets of miRNA-miRNA interactions for different diseases using network inference algorithms on the miRNA-disease expression profiles; the individual predictions of these algorithms were then merged using a consensus-based approach to predict miRNA-miRNA associations. We next selected the miRNA-miRNA associations across particular diseases to generate the corresponding disease-specific miRNA-interaction networks. Next, graph intersection analysis was performed on these networks for multiple diseases to identify the common signature/core sets of miRNA interactions. We applied this pipeline to identify the common signature of miRNA-miRNA inter- actions for cancers. The identified signatures when validated using a manual literature search from *PubMed Central* and the *PhenomiR* database, show strong relevance with the respective cancers, providing an indirect proof of the high accuracy of our methodology. We developed *miRsig*, an online tool for analysis and visualization of the disease-specific signature/core miRNA-miRNA interactions, available at: http://bnet.egr.vcu.edu/miRsig.

MicroRNAs (miRNAs) are non-coding RNAs of ~22 nucleotides in length that inhibit gene expression at the post transcriptional level by binding to the 3′ UTR region of target mRNAs through complementary base pairing^1^. However, a couple of studies have instead reported an activation of target gene expression as well^2^,^3^. By virtue of this gene regulation mechanism, miRNAs play a critical role in several biological processes^4^ and patho-physiological conditions, including cancers^5^. The role of miRNA regulations in diseases have been widely recorded^6^, however the precise patterns through which a miRNA regulates a certain disease(s) are still elusive. For example, it is not yet clear *how* a miRNA’s up/down regulation directly or indirectly affects a disease’s progression or repression because of the many intermediate factors involved. Thus, predicting and identifying miRNA-disease associations has been a primary research area for several groups. Moreover, the multi-level interactions of miRNAs in cancer-like multi-factorial diseases are more complex due to the possibility of several types of interactions, such as, the classical miRNA-mRNA, miRNA-environmental factors, miRNA- transcription factors-miRNA^7^, and our newly hypothesized direct miRNA-miRNA interactions without any intermediate linkers (e.g., transcription factors)^8^. However, till date, no experimental proof of direct miRNA-miRNA interactions exists except, a single study reported in mouse^9^.

Although, the precise patterns or the reasons behind miRNAs’ deregulation in cancers are not fully understood, it has been found that miRNAs tend to work together in groups^10^, as evidenced in certain diseases^11^. Such co-ordinated regulation, comprising mutual co-targeting and co-regulation, as well as miRNA regulation by other miRNAs are reported in many disease conditions, including various cancers^10^. To elucidate the miRNA-disease associations at the regulome level, we earlier developed the *miRegulome* database and corresponding analytic tools^12^. Furthermore, in cancers it has been observed that groups of miRNAs, known as *superfamilies*, express consistently across several cancers and may act as *drivers* of tumorigenesis, where few key miRNAs direct the global miRNA expression patterns^13^. Identification and existence of such groups or super-families of miRNAs obviously leads to the intuition, that the therapeutic suppression or expression of any one of the miRNAs in the family, would compensate for the other participants of the family^13^. Our central hypothesis in this paper is that, these miRNAs in such *superfamilies* may interact directly or indirectly, by forming a core miRNA-miRNA co-regulatory network and thereby acting as a signature component for prognosis, prediction, and early diagnosis of any disease including cancer.

Several computational efforts have been implemented to study and discover the disease-miRNA interaction networks based on functional enrichment analysis^14^, social network analysis methods^15^, similarity-based methods^16^, and diffusion-based methods^17^. Some studies have integrated genomic and phenotype data sets to infer novel miRNA-disease associations^18^. A miRNA regulatory network was also constructed by integrating multidimensional high-throughput data and was used to identify the cancer-associated miRNAs^19^. Similarly, co-regulating miRNA clusters and prioritized candidate miRNAs across multiple types of diseases have been predicted. Using co-regulating functional modules, a miRNA-miRNA synergistic network was constructed to study the aspect of *homophily* among miRNAs associated with the same disease and subsequently disease-specific miRNAs were detected based on their network topological features. In this study, a miRNA-miRNA co-regulation network was constructed by selecting common miRNAs across various data sets related to the same disease, pairing them based on their sharing of common targets, and subsequently performing a GO enrichment analysis of their predicted targets. These miRNAs were qualified as co-regulating if they shared a significant amount of GO enrichment analyses of predicted targets^20^. Disease-specific miRNAs were also identified using the miRNA target-dysregulated network built on the assumption that causative miRNAs show abnormal regulation of their target genes^21^. Similarly, disease-specific miRNAs were also identified by integrating phenotype associations of diseases which had matching miRNA and mRNA expression profiles^22^. Network theoretic algorithms such as the biclique-based method^23^, biclustering technique^24^ and maximum weighted matching^25^ among others have been deployed to discover and predict the patterns of miRNA regulation. Graph theoretical methods and network inference models have also been applied to analyze complex regulatory interactions and reconstruct the causative gene regulatory network and other biological networks^26^,^27^,^28^,^29^.

In this work, we have used the miRNA expression data sets available at the *PhenomiR*^30^ database to predict miRNA-miRNA core/signature interactions across several cancers using a combination of (i) six state-of-the-art network inference algorithms, (ii) a *wisdom of crowds*^31^ based consensus approach^32^ to generate disease-specific miRNA interaction networks with higher accuracy, and (iii) a simplified graph intersection analysis to identify the miRNA-miRNA core interactions across multiple diseases belonging to a particular disease class.

## Methods

**Figure 1:**
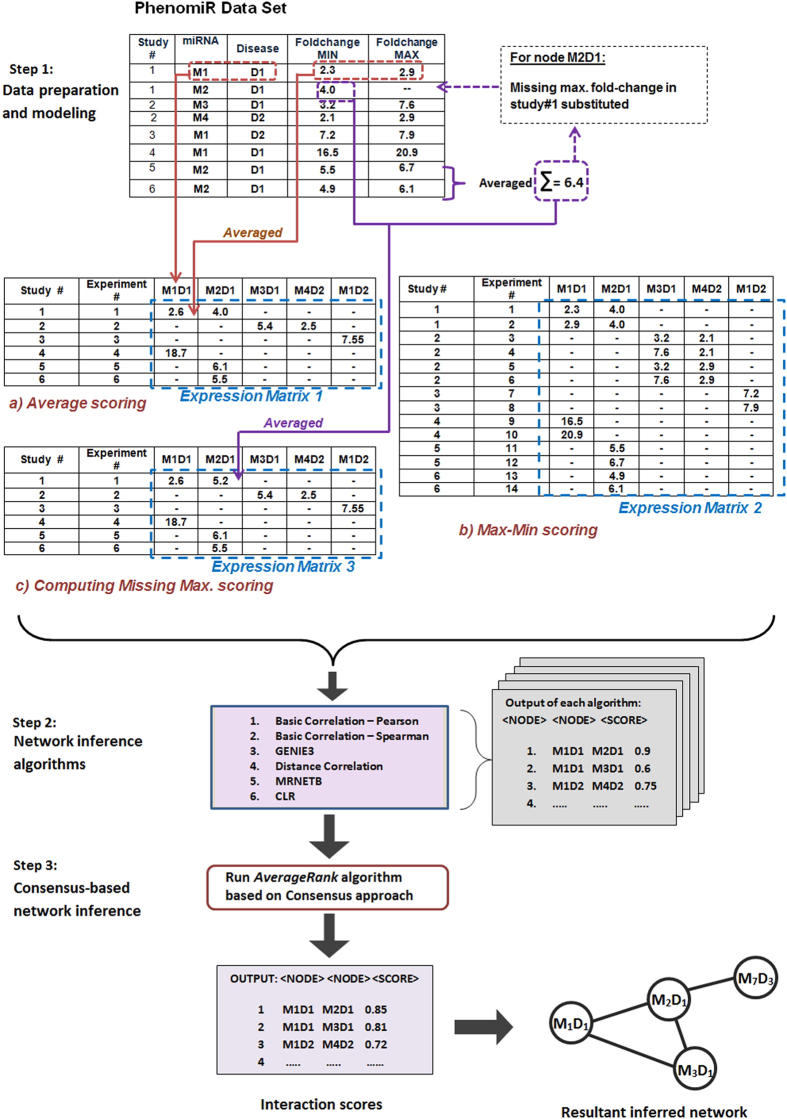
Overview of the methodology with *M*_*i*_ denoting miRNAs and *D*_*j*_ denoting the diseases. *Step 1* consists of translating the *PhenomiR* data set into three miRNA expression matrices (a, b and c) based on three approaches. In *Step 2*, each of these matrices are subjected to six network inference algorithms which produce the interaction scores across the different *M*_*i*_*D*_*j*_ nodes. In *Step 3*, the six individual *M*_*i*_*D*_*j*_ − *M*_*x*_*D*_*y*_ interaction scores are averaged into a final score designating its confidence.

The methodology adopted in this paper is comprised of i) translating the miRNA-disease expression scores from the *PhenomiR* database into a miRNA expression matrix (Fig. 1, Step 1); ii) deploying six network inference algorithms on the expression matrix and deriving the miRNA-miRNA interaction scores from each algorithm (Fig. 1, Step 2); iii) performing a consensus-based approach, i.e. estimating an average score for every miRNA-miRNA interaction across its six predicted scores (Fig. 1, Step 3); iv) validating the resultant interactions using precision-recall analysis with a hypothetical true network generated using the PubMed IDs from *PhenomiR*; v) analyzing the miRNA-miRNA interaction networks for every disease and detection of the conserved miRNA-miRNA interactions across various groups of cancers and finally vi) validating the conserved miRNA-miRNA interactions in the identified group of cancers via manual literature search.

### Data preparation and modeling

**Figure 2:**
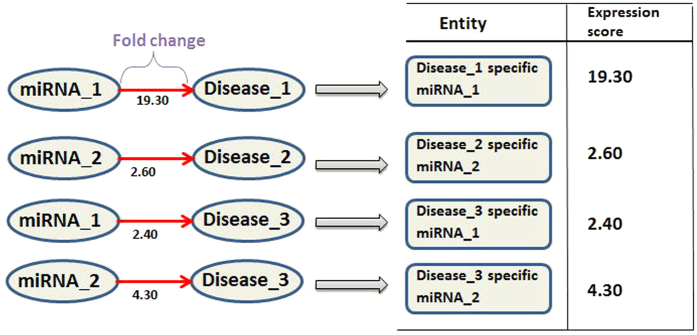
Schematic of the miRNA-disease regulation with fold-change values.

**Figure 3:**
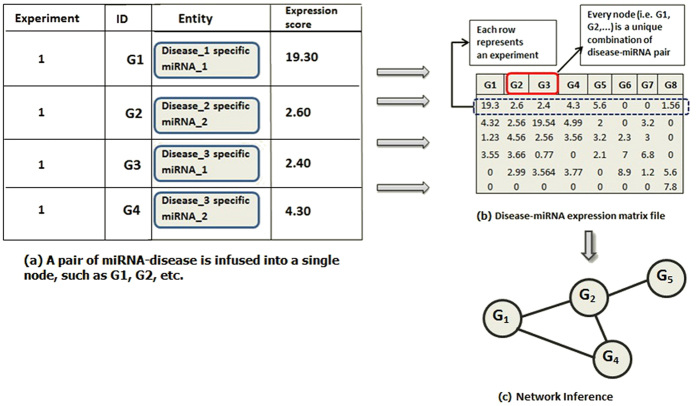
Schematic of the miRNA expression data set. [(**a**) and (**b**)] Data from *PhenomiR* is mapped into an miRNA expression matrix. (**c**) Network inference approach is applied to the matrix to derive the interaction network.

The data from the *PhenomiR* database is freely available and was used in this study. *PhenomiR 2.0* was downloaded for the purposes of this study. *PhenomiR 2.0* is a comprehensive data set containing 535 database entries across 345 articles recording miRNA expressions in diseases^30^. As shown in Fig. 2, the data from *PhenomiR* was converted into a disease-specific miRNA expression matrix (shown in Fig. 3). The miRNAs whose fold-change values were not available in *PhenomiR 2.0* data set were discarded from the study; this also includes some misformatted lines of data that were excluded from further processing as they were also missing the fold-change values. Here, the core idea is to consider a pair of miRNA and disease as a single miRNA-disease (*MD*) node, as seen in Fig. 3; note that, for ease of reference, we consider an *M*_*i*_*D*_*j*_ pair as an *MD* node which conceptually designates a disease-specific miRNA. The same miRNA participating in multiple diseases will have different expression profiles in each of them and hence the disease specific miRNA terminology, i.e., *MD*, signifies a miRNA’s expression profile in a particular disease. Thus, every unique miRNA-disease pair constitutes a unique *MD* type node. In this disease-specific miRNA expression matrix (Fig. 3-b), each row represents a study/experiment and each column represents an *MD*’s expression score in that study. The resultant expression matrix herein, has 4,343 unique nodes/columns (i.e., unique *MD*s in the network) for 267 samples (i.e., rows).

In the *PhenomiR* data set, some *MD*s have two fold-change values indicating minimum and maximum expression scores while other *MD*s only report a minimum fold-change expression score (for e.g., see Fig. 1, Step 1, *PhenomiR* data set, row 2). To assess these scenarios, we devised three different methodologies (described in the next section), generated separate expression matrices based on each methodology and performed the subsequent analysis on each of them.

#### Average scoring

Under *Average scoring* method, for the *MD*s having both minimum and maximum fold-change values per sample, their average was taken and considered as the final expression value in the expression matrix. As shown in Fig. 1, Step-1, the entry *M*1-*D*1 has two expression values - 2.3 and 2.9, i.e., minimum fold-change and maximum fold-change respectively, which were averaged to 2.6 in the *Expression Matrix 1* (see Fig. 1, Step 1-a). For *MD*s with only their minimum expression values reported, this single value was also considered to be its average expression value.

#### Retaining maximum and minimum expression values

The *Average scoring* method can lead to a potential loss of information as the individual maximum and minimum expression values (when available) were not retained. Hence we designed the following two methods to generate the expression matrix.

1. *Max-Min scoring*Under *Max-Min scoring* method, for the *MD*s having minimum and maximum fold-change expression values, (instead of taking their average) both these data points were considered as separate entries; thus, the same *MD* was considered twice in the expression matrix with the duplicate entry designating a new experiment. As displayed in Fig. 1, Step 1, the first row entry, *M*1-*D*1 in *Study-1* has two expression values; these values were individually considered as separate data points and included in the expression matrix accordingly along with their co-expressing miRNAs’ expression values, providing us with *Expression Matrix 2* (see Fig. 1, Step 1-b).
2. *Computing Missing Max. scoring*Under *Computing Missing Max scoring* method, for the *MD*s which did not have a maximum fold-change expression value, we took an average of its maximum fold-change values across all its *other samples* and substituted this average score as it’s maximum fold-change expression value. As shown in Fig. 1, Step 1, the entry *M*2-*D*1 on 2nd row does not have a maximum fold-change value. However, *M*2-*D*1 combination has maximum fold-change expression values of 6.7 and 3.1 from sample #5 and #6, respectively. Herein, we took an average of these two values, i.e. 6.4 and substituted it for the original missing value for *M*2-*D*1 in the 2nd row. This method overcomes the limitation posed due the non-availability of the expression value by giving its closest approximation, based on the particular *MD*’s expression pattern across the sample spectrum. After applying this method, the *Average Scoring* method was performed on this matrix to obtain *Expression Matrix 3* (see Fig. 1, Step 1-c).

After the three expression matrices were derived, a reverse engineering methodology^32^ was adopted to reconstruct the *MD*-*MD* regulatory network from these expression matrices (Fig. 3, *Network Inference*), by applying six widely used network inference algorithms along with a consensus-based ranking algorithm, which is explained in the next section.

### Network inference algorithms

Each expression matrix has 4,343 nodes and therefore, there are potentially 4,343 × 4,343 (i.e. 18,861,649) *MD*-*MD* interactions in the network. Six different network inference algorithms were applied on the miRNA expression matrix, which gave prediction scores for every *MD*-*MD* interaction. We used the mutual information-based algorithm, Context Likelihood of Relatedness (CLR)^33^, Maximum Relevance Minimum Redundancy Backward (MRNETB)^34^, Basic Correlation methods (Pearson and Spearman), Distance Correlation (DC)^35^, and regression-based Gene Network Inference with Ensemble of Trees (GENIE3)^36^ algorithms for network inference. The details of the algorithms are given in Supplementary File S1. Note that, the Basic Correlation methods resulted in two different network inference algorithms based on the type of correlations implemented, i.e., one each for Pearson and Spearman correlations.

### Consensus based network inference approach

**Figure 4:**
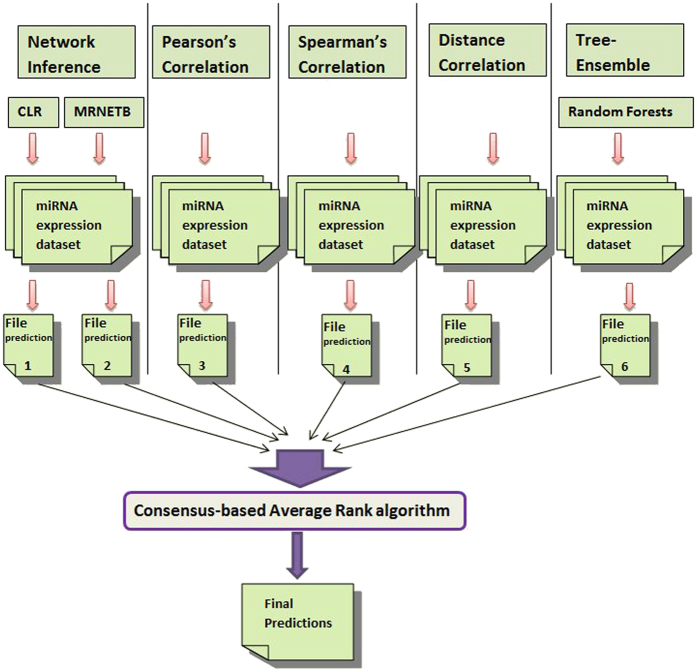
Workflow of the consensus-based miRNA network inference.

Each of the six individual network inference algorithms produced a ranked list of prediction scores for every *MD*-*MD* interaction (see Fig. 1, Step-2). Thereafter, we used the *wisdom of crowds*^31^ approach, which proposes that the aggregation of information from the community yields better results than the individual few. In this study, the consensus based approach aggregates the collective information (i.e. prediction scores) from the six individual network inference algorithms and computes a more accurate final score for *MD*-*MD* interactions. This rank is computed by taking an average of the predicted ranks of each interaction derived from the corresponding network inference algorithms. Figure 4 displays the workflow of this approach. This approach was earlier implemented to infer gene-regulatory networks and yielded highest accuracy compared to each of the individual network inference algorithms^32^.

This consensus based network inference approach is executed in the *Average Rank*^32^ algorithm which essentially computes the average score of a particular *MD*-*MD* interaction by taking the mean of its six predicted ranks. The ranking methodology used in this algorithm is based on the *Borda* count method. This method is used in elections during which voters rank candidates as per their preferences. The winning candidate is the one with the best average rank. Here, all the interactions are first ranked in descending order of their predicted scores (as seen in the column *Rank* in Table 1). Describing briefly, the *Borda* count method allocates points to each rank. The highest ranked interaction (meaning, 1) get the maximum *Borda* points (number of interactions - 1) and the lowest ranked interaction has 0 *Borda* points as demonstrated in the column *Borda points* in Table 1. In order to derive the final rank between 0 and 1, these points are thereafter normalized to derive a relative *Borda* rank. Thus, each rank has been translated to its new relative *Borda* rank. Note that, the *Borda* count ranking method is among the many other methods to perform *averaging* of the ranks in the consensus methodology.

The six network inference algorithms generate six different ranks for each interaction and the consensus algorithm next computes an average *Borda* rank for the interaction. Tables 1 and 2 display a scenario of ranking four *MD*-*MD* interactions *I*_1_, *I*_2_, *I*_3_ and *I*_4_ via a consensus-based approach as executed in *AverageRank* algorithm. Table 1 displays the ranked list of predictions for these interactions by all the six network inference algorithms based on their prediction scores. For example, in Table 1, *Algorithm 1* ranks *MD*-*MD* interactions in this order — *I*_4_, *I*_2_, *I*_1_ and *I*_3_ based on their prediction scores. The individual ranks for miRNA-miRNA interaction *I*_4_ are 1, 3, 4, 2, 2 and 3 by the six algorithms respectively (noted with *), and their relative respective *Borda* ranks are 1, 0.333, 0, 0.666. 0.666 and 0.333. The final rank of interaction *I*_4_ is the average of all the *Borda* ranks, i.e., 0.49, as demonstrated in Table 2 (noted with *). Similarly the final ranks of every other interaction is computed using the following formula,


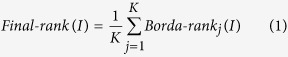


where, *K* is the number of algorithms (six, in our case). These results are displayed in Table 2.

An example of the final result listing of our *MD-MD* interactions is shown in Table 3 (also see Fig. 1 Step-3).

In these results, we noted all the different possibilities of interactions that can occur considering the miRNA-disease pair, i.e. *MD* as a node. There are essentially four types of interactions that can exist in this network. These are explained in Table 4. Among these types, *type 1* is a self-loop and not applicable for our purposes. For application purposes of our methodology, we focused on analyzing the set of interactions belonging to *type 3* which is further elaborated in the next section. Interactions of *type 2* and *type 4* will be studied in the future to analyze the relationship between diseases sharing a common miRNA (*type 2*) and the proximity between dissimilar miRNAs and dissimilar diseases (*type 4*) having high probabilities of interaction.

### Disease-specific miRNA network construction

**Figure 5:**
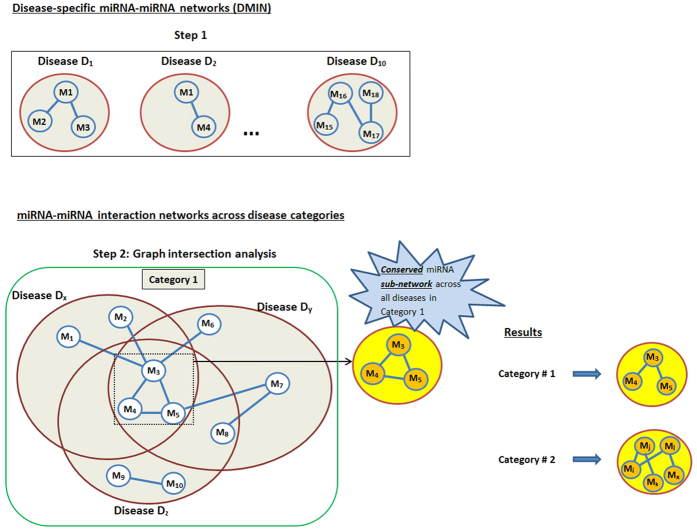
Overview of the disease analysis.

In this section, the results of the *type 3* interactions were selected for disease-specific analysis. There were 66 unique diseases in the final predicted list of interactions from the *Average Rank* algorithm; this list of diseases are provided in the Supplementary File S2. Under a specific disease *D*_*x*_, all the miRNA-miRNA edges, i.e. *M*_1_*D*_*x*_ − *M*_2_*D*_*x*_ edges were collected into a single *D*_*x*_ disease network; thereby giving us the **d**isease-specific **m**iRNA-miRNA **i**nteraction **n**etwork (*DMIN*) (Fig. 5, Step 1). *DMIN* is a network *G* = (*V, E*), where *V* = {*M*_1_*D*_*x*_, *M*_2_*D*_*x*_, … *M*_*n*_*D*_*x*_} (i.e., set of miRNAs under disease name *D*_*x*_) and *E* is the ordered set of edges, where edge *e* = {*M*_*i*_, *M*_*j*_}. We performed a similar network construction for every cancer-related disease, *D*_*x*_. To pursue a more definitive and cancer-specific analysis, only cancer-related diseases were chosen and grouped into classes based on their tissue/organ specificity. We created four major classes: i) gastrointestinal cancers (esophageal, gastroesophageal, gastrointestinal, gastric, and colorectal cancer), ii) endocrine cancers (hepatocellular, pancreatic, and thyroid carcinoma follicular, and thyroid carcinoma papillary), iii) leukemia/blood cancers (hematological tumors, acute myeloid leukemia, chronic lymphatic leukemia, and acute myelogenous leukemia), and iv) nerve cancers (neuroblastoma, medulloblastoma, and glioblastoma).

Under a particular disease class, all the corresponding *DMIN*s were combined into a single network (Fig. 5, Step 2). Using graph intersection analysis, we mined the miRNA-miRNA interaction networks of all the cancers within the specific class to identify a conserved (signature/core) miRNA-miRNA interaction component. This identified miRNA-miRNA interaction component was present in all the diseases of that particular class. These findings are reported in the *pan-cancer miRNA signatures* section and the results are discussed in the *Discussion* section.

## Results

### Validation of interactions

After executing the *Consensus based network inference approach* on three input miRNA expression matrices derived from the three approaches mentioned in the *Data preparation and modeling* section (*Average scoring, Retaining Max-Min* and *Computing Missing Max.*), we obtained three sets of predicted miRNA-miRNA interactions. Each predicted interaction was validated by querying for PubMed IDs in the *PhenomiR* database which cited and reported the occurrence of miRNAs’ association with the specific disease in a single PubMed ID. For e.g., for each predicted interaction, i.e. *M*_*a*_*D*_*x*_ to *M*_*b*_*D*_*x*_, if a PubMed ID cited the occurrence of the association between the miRNAs (*M*_*a*_, *M*_*b*_) and the disease (*D*_*x*_), the interaction was termed as true/validated (1); else the predicted interaction was termed as unknown/unverified (0). Based on this, labels were generated for every interaction in the resultant set forming the true network. We performed a precision-recall analysis to ascertain the accuracy of the consensus-based network inference method. The precision-recall values were calculated using the formula:


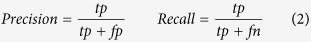


where *tp, fp*, and *fn* are true-positives, false-positives and false-negatives respectively.

**Figure 6:**
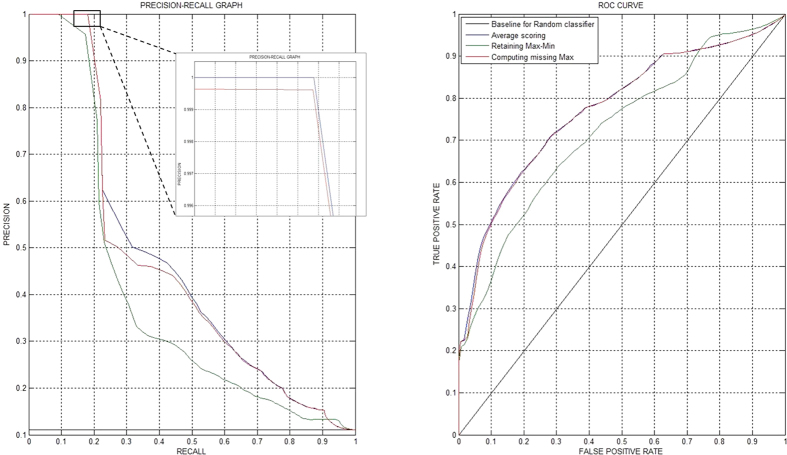
Precision-recall and ROC curves displaying the accuracy of the three methods. The figure demonstrates that the *Average scoring* (blue curve) method fared better than *Retaining Max-Min* (green curve) and *Computing Missing Max.* (red curve) methods. The inset image shows that the precision of *Average scoring* method slightly outperformed the *Computing Missing Max.* and was the best overall performer.

Figure 6 displays the results of the precision-recall analysis and the ROC curve for all the three approaches used. As demonstrated in the figure, the *Average scoring* method fared better than the other two methods; in fact the *Computing Missing Max.* method also performed well for low recall but gradually degraded for higher recall values. Based on this precision-recall curve, our proposed methodology displays a high precision (for up to a 30% recall) demonstrating its effectiveness in providing high confidence to the results. The ROC curve shows that both the *Average scoring* and *Computing Missing Max.* methods are comparable in predicting the true positives when compared to the number of false positives seen alongside.

Note that our true network generation method has some obvious limitations. While a true edge constituting the association of the two miRNAs with the same disease in the same PubMed ID is still acceptable (specifically because these edges were manually curated), the unverified edges may simply mean that a study has not yet been reported associating the miRNAs to the same disease. Hence, a high precision performance should be the best judge of our methodology whereas the recall curve can be somewhat circumstantial.

### Pan-cancer miRNA signatures

**Figure 7:**
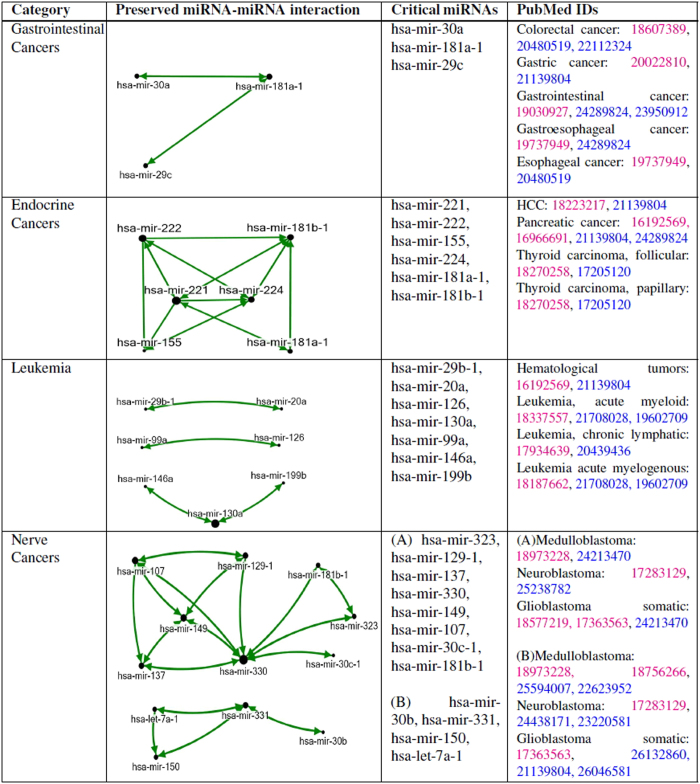
Signature miRNA-miRNA interaction component identified in various cancer categories. The PubMed IDs citing the critical miRNAs with the disease from the *PhenomiR* database are in magenta while the PubMed IDs from the *PubMed Central* database are in blue.

After the *Validation of interactions*, in order to confidently detect miRNA signatures in the specified disease classes, only the top 10% interactions with the highest confidence scores were used in the construction of *DMIN* (Fig. 5, Step 1) and the subsequent graph intersection approach (Fig. 5, Step 2). Hence, all the considered miRNA-miRNA interactions had a confidence score of 0.9 and above. As reported in Fig. 7, under gastrointestinal cancers, we detected a signature component of three miRNAs (hsa-mir-30a, hsa-mir-181a-1, and hsa-mir-29c). For endocrine cancers, the signature component consisted of hsa-mir-221, hsa-mir-222, hsa-mir-155, hsa-mir-224, hsa-mir-181a-1, and hsa-mir-181b-1. For leukemia cancers, the signature component consisted of hsa-mir-29b-1, hsa-mir-106a, hsa-mir-20a, hsa-mir-126, and hsa-mir-130a. We observed two different signatures for nerve cancers. For subsequent validation of these cancer-specific signature set of miRNAs, we manually mined PubMed articles which corroborate our results, as reported in Fig. 7. We queried both the *PhenomiR* database and the *PubMed Central* database for these reported PubMed IDs; the results from these two sources are shown in different colors in Fig. 7. We also observed that, while hsa-mir-30 is common in gastrointestinal and nerve cancers; hsa-mir-181 is shared by gastrointestinal, endocrine and nerve cancers. The miRNA signature component of the category leukemia is found to possess a distinct group of miRNAs (Fig. 7). The role and involvement of these miRNAs in their associated diseases are further elaborated in the *Discussion* section.

**Figure 8:**
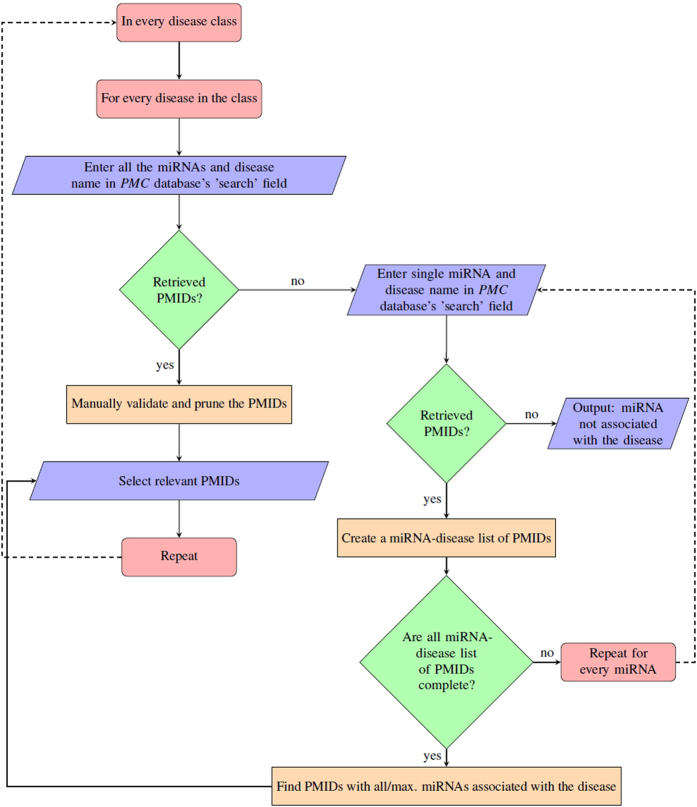
Flowchart of the workflow for manual literature search.

The individual steps involved in the manual search process from *PubMed Central* are shown in Fig. 8. To summarize, we first searched *PubMed Central* with the list of core miRNAs and each disease for which they form a signature component. We next manually checked the ‘search’ results to confirm the associations (i.e., the pruning step for PMIDs). If not enough results were retrieved from this search, we entered each miRNA, disease pair individually for all the miRNAs forming the signature component in that disease; each of these results were then manually pruned and collated to give us the set of PMIDs corresponding to the core miRNAs for that disease. This process was repeated for all the other diseases of a particular disease class.

### *miRsig* - an online tool

**Figure 9:**
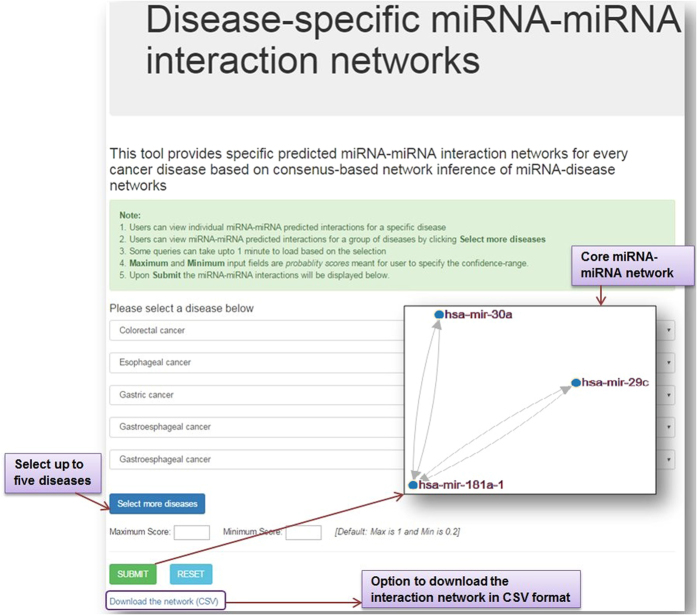
miRNA-miRNA interactions shown in *miRsig* for esophageal, gastroesophageal, gastrointestinal, gastric, and colorectal cancers.

In order to aid researchers to identify disease-specific miRNA-miRNA interaction networks across several diseases, we developed the *miRsig* tool, available at http://bnet.egr.vcu.edu/miRsig. *miRsig* allows the user to visualize the miRNA-miRNA interaction network for each disease recorded in *PhenomiR* and also across multiple diseases. The results are based on the consensus-based network inference approach. *miRsig* also allows users to search for a common/core miRNA-miRNA interaction component in a user-specified selection of diseases (see Fig. 9). Users can create their own class/category of cancers by *selecting* more diseases, as shown in Fig. 9. The edges in the interaction have confidence scores as weights, from 0 (minimum) to 1 (maximum). Hence, the tool also allows the user to view only the higher/lower/specific confidence interactions by changing the *Maximum* and *Minimum* confidence score ranges. Currently, the total number of edges across the entire miRNA-miRNA interaction networks are more than 18 million. Hence, to avoid cluttering of the result set and to allow clear visibility and comprehension of the network, the *Minimum* score is set to 0.5, if not specified by the user. Users can also view and analyze the topological properties of miRNA clusters interacting in each or a set of diseases. The signature/core miRNA-miRNA interactions among esophageal, gastroesophageal, gastrointestinal, gastric, and colorectal cancers, as predicted and visualized is shown in Fig. 9. This network component consisting of three miRNAs (has-mir-30a, has-mir-181a-1, and has-mir-29c) is the signature component for all the aforementioned five cancers, and can be validated using simple literature search on *PubMed Central* database as demonstrated in Fig. 8.Users can also download the miRNA-interaction network in the format of an edge-list in a CSV file. This edge-list can be imported in various network analysis tools such as, *NodeXL, Cytoscape*, etc. for further study and analysis of the interaction network.

*miRsig* tool has been developed using MySQL as the back-end database and HTML, PHP, JavaScript, AJAX for front-end design. The interactive network visualization has been implemented using data visualization library, D3.js^37^.

## Discussion

miRNA-mRNA interactions have been substantially documented^38^ and is a prime area of ongoing research. Similarly, miRNA- miRNA interactions through mutual co-expression^39^, via transcription factor^40^, and miRNA-disease associations^6^ have also been reported. However, miRNA-miRNA interactions towards identification of a core miRNA-miRNA module that could potentially be a signature component for a particular disease have not been studied enough. Many studies have used computational approaches to study this aspect. A miRNA-miRNA co-regulation network in lung cancer was identified using a progressive data refining approach^20^. Similarly, miRNA expression profiling along with a genome-wide SNP approach was used to create a miRNA-miRNA synergistic network to study coronary artery disease^41^. miRNA-miRNA interactions were also identified in esophageal cancer using K-clique analysis on a bipartite network consisting of miRNAs and subpathways^42^. Additionally, miRNA-target interactions were integrated with miRNA and mRNA expressions to deduce miRNA-miRNA interactions in prostate cancer^43^. A network topological approach was also undertaken to identify disease miRNAs by constructing a miRNA-miRNA synergistic network consisting of co-regulating functional modules^44^.

In this work, we adopted a strategy that takes a miRNA expression profile and uses six different network inference algorithms (CLR^33^, MRNETB^34^, Basic Correlation (Pearson and Spearman), DC^35^, GENIE3^36^), each varying in their inference strategies, integrated with a consensus approach and graph intersection to identify the conserved miRNA-miRNA interaction signature across a group of diseases (cancers, in this case). The identified signatures were validated via manual literature search and were found to be associated within the classes of the selected cancers, demonstrating the efficacy of the method. Under validation, we retrieved the PMIDs reporting the associations from the *PhenomiR* database and also performed a manual literature search in the *PubMed Central* database to separately corroborate our results, as displayed in Fig. 7.

Our results show that, the expression profile of hsa-mir-30a, hsa-mir-181a-1, and hsa-mir-29c could be a signature for gastrointestinal cancers that comprises of esophageal, gastroesophageal, gastrointestinal, gastric, and colorectal cancers (Fig. 7). These miRNAs are already reported to be associated with these cancers^45^,^46^,^47^,^48^. miRNAs (hsa-mir-30a, hsa-mir-29c, hsa-mir-181a-1) displayed the same trend of expression in a study of esophageal adenocarcinoma (EAC) and Barrett’s esophagus (BE) and were differentially up-regulated in both the disease tissues. hsa-mir-181a and hsa-mir-29c showed higher expression levels in EAC to that of BE with high grade dysplasia^48^. Studies have also reported hsa-mir-181a, hsa-mir-30a and hsa-mir-29c being overexpressed in esophagela carcinoma (EC) and hsa-mir-29c to be underexpressed in EC^49^,^50^ and therefore, this group of miRNAs may be considered for developing a pan-diagnostic tool for the aforementioned cancers.

We identified that hsa-mir-221, hsa-mir-222, hsa-mir-155, hsa-mir-224, hsa-mir-181a-1, and hsa-mir-181b-1 make the signature for endocrine cancers (hepatocellular, pancreatic, and thyroid cancers) (Fig. 7). Reports suggest that these miRNAs are predominantly associated with this group of cancers^51^,^52^,^53^,^54^. In another study analyzing molecular signatures for aggressive pancreatic cancer, all the miRNAs (hsa-mir-221, hsa-mir-222, hsa-mir-155, hsa-mir-224, hsa-mir-181a-1, and hsa-mir-181b-1) were significantly altered due to chronic exposure to conventional anti-cancer drugs^55^. A large-scale meta-analysis investigating candidate miRNA biomarkers for pancreatic ductal adenocarcinoma (PDAC) across eleven miRNA expression profiling studies, reported all the miRNAs to be up-regulated and having a consistent direction of change. miRNAs hsa-mir-221, hsa-mir-222, hsa-mir-155 were reported to be upregulated together in at least five of these studies with a consistent direction. Among them, miRNAs hsa-mir-221, hsa-mir-155 were identified as part of a meta-signature and biomarkers for PDAC^56^. Studies also report all these miRNAs to be associated with lung cancer^57^. Thus this set of miRNAs may be used/tested as a diagnostic tool for all the endocrine cancers considered here.

Seven miRNAs (hsa-mir-29b-1, hsa-mir-146a, hsa-mir-20a, hsa-mir-126, hsa-mir-99a, hsa-mir-199b and hsa-mir-130a) that are well documented for their association with various kinds of leukemia^54^,^58^,^59^,^60^,^61^,^62^,^63^ are found to form the signature component of leukemia from our analysis (Fig. 7). miRNAs (hsa-mir-29b-1, hsa-mir-20a, hsa-mir-126, hsa-mir-146a, hsa-mir-199b) were differentially expressed in a blood stem cell study in which the blood stem cells were treated with plerixafor and granulocyte colony-stimulating factor. The miRNAs were recorded to be expressed in this treated cell study analyzing acute lymphocytic leukemia conditions^64^. miRNAs (hsa-mir-126, hsa-mir-130a, hsa-mir-99a, hsa-mir-146a, hsa-mir-199b) have also been reported to express together in a myeloid cell study exploring transcription factor binding site motifs^65^. Therefore, this signature group of miRNAs can be potentially used as a screening or diagnostic tool for a range of different types of leukemia.

In case of neurone cancers (neuroblastoma, medulloblastoma, and glioblastoma) we detected two signatures: i) hsa-mir-323, hsa-mir-129-1, hsa-mir-137, hsa-mir-330, hsa-mir-149, hsa-mir-107, hsa-mir-30c-1, hsa-mir-181b-1 and ii) hsa-mir-30b, hsa-mir-331, hsa-mir-150, hsa-let-7a-1 (Fig. 7). Regarding the first signature network component, hsa-mir-137, hsa-mir-330, hsa-mir-149, hsa-mir-107, hsa-mir-181b were among the miRNAs whose experimentally validated targets (such as CTBP1, CDC42, CDK6, E2F1, VEGFA, AKT1, KAT2B) affect the pathways which play a crucial role in glioblastoma biology. Deregulations of hsa-mir-137, hsa-mir-330 and hsa-mir-149 lead to effects in the glioma de novo pathway, VEGF signaling pathway and Notch signaling pathway^66^. Among the miRNAs reported in the second signature component, hsa-mir-330 and hsa-mir-30b are among the top ten miRNAs having least coefficient of variation in the expression of benign kidney tumor and hsa-mir-150 is differentially expressed in metastatic clear cell renal cell carcinoma^67^.

Comparing our results with other similar works has been challenging, primarily because there are not many studies that have reported direct miRNA-miRNA co-regulations across these disease classes. Similar studies^13^,^20^,^68^ have used different disease and miRNA data sets which makes a one-to-one comparison challenging. In some previous works, miRNA-miRNA regulatory associations have been deduced based on the semantic similarities between the associated diseases^69^ and based on the analysis of shared transcription factors, common targets, KEGG pathway analysis and corroboration from literature^20^. However, none of these methods allow for a network-level miRNA-miRNA analysis for a variety of diseases and hence cannot be used for comparison purposes to the predicted interaction networks in this paper.

Online analysis and visualization of results is an aid to the research community. Along these lines, several network analysis and visualization tools have been developed, such as *VisANT* for integrative online visual analysis of biological networks and pathways^70^, *miRegulome* for miRNA regulome visualization and analysis^12^ and *miRNet* for functional analysis of miRNAs within a high-performance network visual analytics system^71^ among others. However, no tool is available so far which can perform an online visualization and analysis of signature miRNAs across multiple diseases. The *miRsig* tool developed here bridges this gap and provides an intuitive analysis and visualization of core/signature miRNA-miRNA interaction components for several diseases.

## Conclusion

In this work, we have developed a novel consensus-based network analysis pipeline to identify disease-specific miRNA-miRNA interactions by combining the expression profiles of various miRNAs in specific diseases. This method can effectively identify the signature/core miRNA-miRNA interactions for a group of diseases; here tested on cancer. These signature miRNAs may have potential use for diagnostic, prognostic, or therapeutic applications for a group of related diseases such as cancers. The predicted miRNA-miRNA signature patterns were extensively validated by the PMIDs reported in the *PhenomiR* database as well as an independent manual literature search from *PubMed Central. miRsig* thus provides a powerful prediction and visualization tool for the identification of core/signature miRNA-miRNA interactions amongst a number of diseases. Our future work includes investigating the (i) *miRNA*_*same*_*Disease*_*different*_ category of interactions to study the dynamics of similar miRNAs across multiple diseases and also (ii) the *miRNA*_*different*_*Disease*_*different*_ category of interactions to understand the evolution of diseases based on the underlying miRNA expression patterns. As miRNAs may potentially serve as biomarkers for a wide variety of diseases, our proposed pipeline may motivate the study of several interesting questions both for particular diseases or across multiple diseases.

## Additional Information

**How to cite this article**: Nalluri, J. J. *et al. miRsig*: a consensus-based network inference methodology to identify pan-cancer miRNA-miRNA interaction signatures. *Sci. Rep.* **7**, 39684; doi: 10.1038/srep39684 (2017).

**Publisher's note:** Springer Nature remains neutral with regard to jurisdictional claims in published maps and institutional affiliations.

## Supplementary Material

Supplementary Information

Supplementary Data

## Figures and Tables

**Table 1:** Ranked individual predictions of each algorithm for every interaction *I*.

*Borda rank* (Norm. borda points)	Borda points	Rank	Alg. 1	Alg. 2	Alg. 3	Alg. 4	Alg. 5	Alg. 6
1	3	1	*I*_4_^*^	*I*_2_	*I*_2_	*I*_2_	*I*_2_	*I*_3_
0.6667	2	2	*I*_2_	*I*_3_	*I*_3_	*I*_4_^*^	*I*_4_^*^	*I*_2_
0.3334	1	3	*I*_1_	*I*_4_^*^	*I*_1_	*I*_3_	*I*_3_	*I*_4_^*^
0	0	4	*I*_3_	*I*_1_	*I*_4_^*^	*I*_1_	*I*_1_	*I*_1_

**Borda points** are allocated to each **Rank**. A relative ***Borda rank*** 
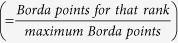
 is computed for every **Rank**. *Borda* ranks for interaction *I*_4_ (noted with *) are 1, 0.333, 0, 0.666, 0.666 and 0.334 by the six algorithms respectively.

**Table 2:** Final ranks for each interaction; the final rank of interaction *I*_4_ is 0.49.

Interaction	Averaging of *Borda* ranks	Final rank
*I*_2_	(0.66 + 1 + 1 + 1 + 1 + 0.66)/6	0.88
*I*_3_	(0 + 0.66 + 0.66 + 0.33 + 0.33 + 1)/6	0.49
*I*_4_^*^	(1 + 0.33 + 0 + 0.66 + 0.66 + 0.33)/6	0.49^*^
*I*_1_	(0.33 + 0 + 0.33 + 0 + 0 + 0)/6	0.11

**Table 3:** Format of the results based on the consensus approach.

Rank	Interaction	Score
1	Hepatocellular carcinoma:hsa-mir-183 ⇒ Hepatocellular carcinoma:hsa-mir-374a	0.9786
2	Hepatocellular carcinoma:hsa-mir-374a ⇒ Hepatocellular carcinoma:hsa-mir-182	0.9781
3	Breast cancer:hsa-let-7a-1 ⇒ Breast cancer:hsa-mir-30d	0.2985
4	Breast cancer:hsa-let-7a-1 ⇒ Breast cancer:hsa-mir-381	0.2426

**Table 4:** Types of interactions in the network.

Type #	Interaction type	Edge	Remark
1	*miRNAs*_*same*_, *Diseases*_*same*_	*M*_1_*D*_1_ → *M*_1_*D*_1_	Self-loops, N/A
2	*miRNAs*_*same*_, *Diseases*_*different*_	*M*_1_*D*_1_ → *M*_1_*D*_2_	Present in the result set
3	*miRNAs*_*different*_, *Diseases*_*same*_	*M*_1_*D*_1_ → *M*_2_*D*_1_	Present and used for analysis
4	*miRNAs*_*different*_, *Diseases*_*different*_	*M*_1_*D*_1_ → *M*_2_*D*_2_	Present in the result set
