# Supplementary material for: miRsig: a consensus-based network inference methodology to identify pan-cancer miRNA-miRNA interaction signatures
Source: Sci Rep. 2017 Jan 3;7:39684. doi: 10.1038/srep39684 (PMC5206712; doi:10.1038/srep39684)
Supplement: Supplementary Information [file srep39684-s1.pdf]

SUPPLEMENTARY FILE - S1

miRsig: a consensus-based network inference methodology to identify pan-cancer miRNA-miRNA interaction signatures

Joseph J. Nalluri[1,*], Debmalya Barh[2], Vasco Azevedo[3] and Preetam Ghosh[1]

1. Department of Computer Science, School of Engineering, Virginia Commonwealth University, Richmond, Virginia, USA
2. Center for Genomics and Applied Gene Technology, Institute of Integrative Omics and Applied Biotechnology, Purba Medinipur, West Bengal, India
3. Laboratório de Genética Celular e Molecular, Departamento de Biologia Geral, Instituto de Ciências Biológicas (ICB), Universidade Federal de Minas Gerais, Pampulha, Belo Horizonte, Minas Gerais, Brazil

*Corresponding author: nallurijj@mymail.vcu.edu

SUPPLEMENTARY FILE- S1

Details of the Network Inference Algorithms used in this work

1. CLR

Context likelihood of relatedness (CLR)[1] algorithm belongs to the class of relevance networks. Relevance network algorithms use mutual information (MI) based Z scores between a regulator-target pair to identify and determine potential interactions between them. If the MI score is above a certain threshold among the expression dataset, the interaction between the pair is highly likely. In the past, CLR method has been proven to be effective in learning novel transcriptional interactions in E. coli.[1] CLR uses the metric of MI to gauge the similarity between the expression profiles of two entities, in our case, disease-specific miRNAs. The MI is calculated as below:

$$I(X;Y) = \sum_{i,j} P(x_i, y_i) \log \frac{p(x_i, y_i)}{p(x_i)p(y_i)}$$

where, X and Y are random variables i.e. two miRNAs in this case. $P(x_i)$ denotes the probability of X = x_i. MI values between a $miRNA_i$ and $miRNA_j$are calculated and thereafter estimated with regards to the likelihood of their occurrence by comparing that score with a background null model, which is the distribution of MI values. The null model incorporates two sets of MI values: MI_i which is a set of all $miRNA_i$'s MI values and MI_j, a set of all MI values of $miRNA_j$. These two sets, MI_i and MI_j are two independent variables used in the computation of the joint distribution of the null-model, i.e. the background MI. Thus the MI score of the pair($miRNA_i$,$miRNA_j$) is compared with two Z scores resulting from MI_i and MI_j. Further in-depth explanation of the algorithm can be found in[1]. However, CLR predominantly relies on the MI matrix for its scores, and cannot ascertain for causality between the regulators which is more often based on the regulatory kinetics exhibited in the time-series data[2]

The CLR algorithm, available in the ‘minet’ R Bioconductor package[3] was used in this work.

2. GENIE3

Gene Network Inference with Ensemble of Trees (GENIE3)[4] algorithm was the top performing algorithm in the 'DREAM4- In Silico Network Challenge'[5] of inferring gene regulatory network. GENIE3 has a different approach of inferring the associations by taking into account, feature selection as compared to CLR, which is mutual-information based. In GENIE3, a regression problem is formulated for each individual miRNA. Hence in our case, 4343 regression problems were solved for each disease-specific miRNAs. In this regression analysis, each disease-specific miRNA's expression pattern is predicted from every other miRNA's expression pattern by the application of 'Random Forests'[6] model which is a tree-based ensemble model. Thus, based on the significance of the expression patterns between two disease-specific miRNAs and the least output variance between the target miRNA and the considered miRNA, a regulatory link between them is predicted. In this way, the algorithm ranks all the interactions between the miRNAs based on their aggregated scores from regression analysis. GENIE3 is adaptable to other categories of expression data involving interactions.

The GENIE algorithm hosted on GenePattern[7] was executed with standard run conditions - tree-based method as 'Random Forests' and the number of trees grown in an ensemble as 500.

3. Basic Correlation

The Basic Correlation method ranks every disease-specific miRNA-miRNA pair according to the correlation between them. This algorithm uses the Pearson's and Spearman's coefficient to calculate their correlation score,

Pearson's coefficient:

$$\rho_{X,Y} = corr(X,Y) = \frac{cov(X,Y)}{\sigma_X \sigma_Y} = \frac{E[(X-\mu_X)(Y-\mu_Y)]}{\sigma_X \sigma_Y}$$

where,

X and Y are random variables, with μ_X and μ_Y being the expected values and σ_X and σ_Y being the standard deviations. The Spearman correlation coefficient

$$\rho = 1 - \frac{6\sum {d_i}^2}{n(n^2-1)}$$

where, d_i is the difference between the ranks of corresponding values X_i and Y_i and n is the number of points in dataset. While running the Basic Correlation algorithm hosted on the tool GenePattern[7] the ranks of the disease-specific miRNA-miRNA were derived using Pearson's correlation coefficient and Spearman's correlation. Note that, there are two resultant files - one with Pearson's correlation coefficient and the other with Spearman's correlation coefficient, under this approach.

4. **MRNETB**

MRNETB[8] is a mutual information based network inference algorithm which is an improved version of its predecessor MRNET. MRNET performs network inference using the method 'Maximum Relevance Minimum Redundancy (MRMR)'. For every random variable X_i, a set of predictor variables are chosen based on the difference of mutual information between X_i and the set of variables $X_i \in X_{S_j}$. This method follows forward selection, i.e. a variable X_i with highest MI score with the target variable X_j is chosen at first. Hence, the general idea behind the algorithm is identification of subsets of X_{S_j} for every variable with which the variable in the set has maximum pairwise relevance and maximum pairwise independence. While previous methods recursively select a subset of variables and compute the mutual information scores, MRNETB performs backward elimination with sequential search. Further, in-depth explanation can be found at[8].

This algorithm was run from the Bioconductor package 'minet'[3] with Spearman entropy estimator and the number of bins used for discretization was $\sqrt{N}$, where N=number of samples, i.e. 267 in our expression dataset.

5. **Distance Correlation**

This algorithm is described in[9] which uses a novel measurement of dependence called distance correlation (DC)[10] to derive non-linear dependencies from the gene expression dataset. This metric uses a different approach as compared to some of the

previous MI based methods. MI based methods rely on density estimator of certain patterns which can be challenging for multivariate data, it cab be challenging. Also, in the case of continuous data, it has to be discretized before MI based methods are applied. The algorithm details can be found in [9] and beyond the scope of this work.

References

1. Faith JJ, Hayete B, Thaden JT, et al. Large-scale mapping and validation of Escherichia coli transcriptional regulation from a compendium of expression profiles. Levchenko A, ed. *PLoS Biol*. 2007;5(1):e8. doi:10.1371/journal.pbio.0050008.
2. Madar A, Greenfield A, Vanden-Eijnden E, Bonneau R. DREAM3: network inference using dynamic context likelihood of relatedness and the inferelator. *PLoS One*. 2010;5(3):e9803.
3. Meyer PE, Lafitte F, Bontempi G. minet: AR/Bioconductor package for inferring large transcriptional networks using mutual information. *BMC Bioinformatics*. 2008;9(1):461.
4. Irrthum A, Wehenkel L, Geurts P, others. Inferring regulatory networks from expression data using tree-based methods. *PLoS One*. 2010;5(9):e12776.
5. Dream Challenges.
6. Breiman L. Random forests. *Mach Learn*. 2001;45(1):5-32.
7. Reich M, Liefeld T, Gould J, Lerner J, Tamayo P, Mesirov JP. GenePattern 2.0. *Nat Genet*. 2006;38(5):500-501.
8. Meyer P, Marbach D, Roy S, Kellis M. Information-Theoretic Inference of Gene Networks Using Backward Elimination. In: *BIOCOMP*. ; 2010:700-705.
9. Guo X, Zhang Y, Hu W, Tan H, Wang X. Inferring Nonlinear Gene Regulatory Networks from Gene Expression Data Based on Distance Correlation. *PLoS One*. 2014;9(2):e87446.
10. Székely GJ, Rizzo ML, Bakirov NK, others. Measuring and testing dependence by correlation of distances. *Ann Stat*. 2007;35(6):2769-2794.
